# Supplementary figures and images for: The thalamus encodes and updates context representations during hierarchical cognitive control
Source: PLoS Biol. 2024 Dec 2;22(12):e3002937. doi: 10.1371/journal.pbio.3002937 (PMC11637348; doi:10.1371/journal.pbio.3002937)

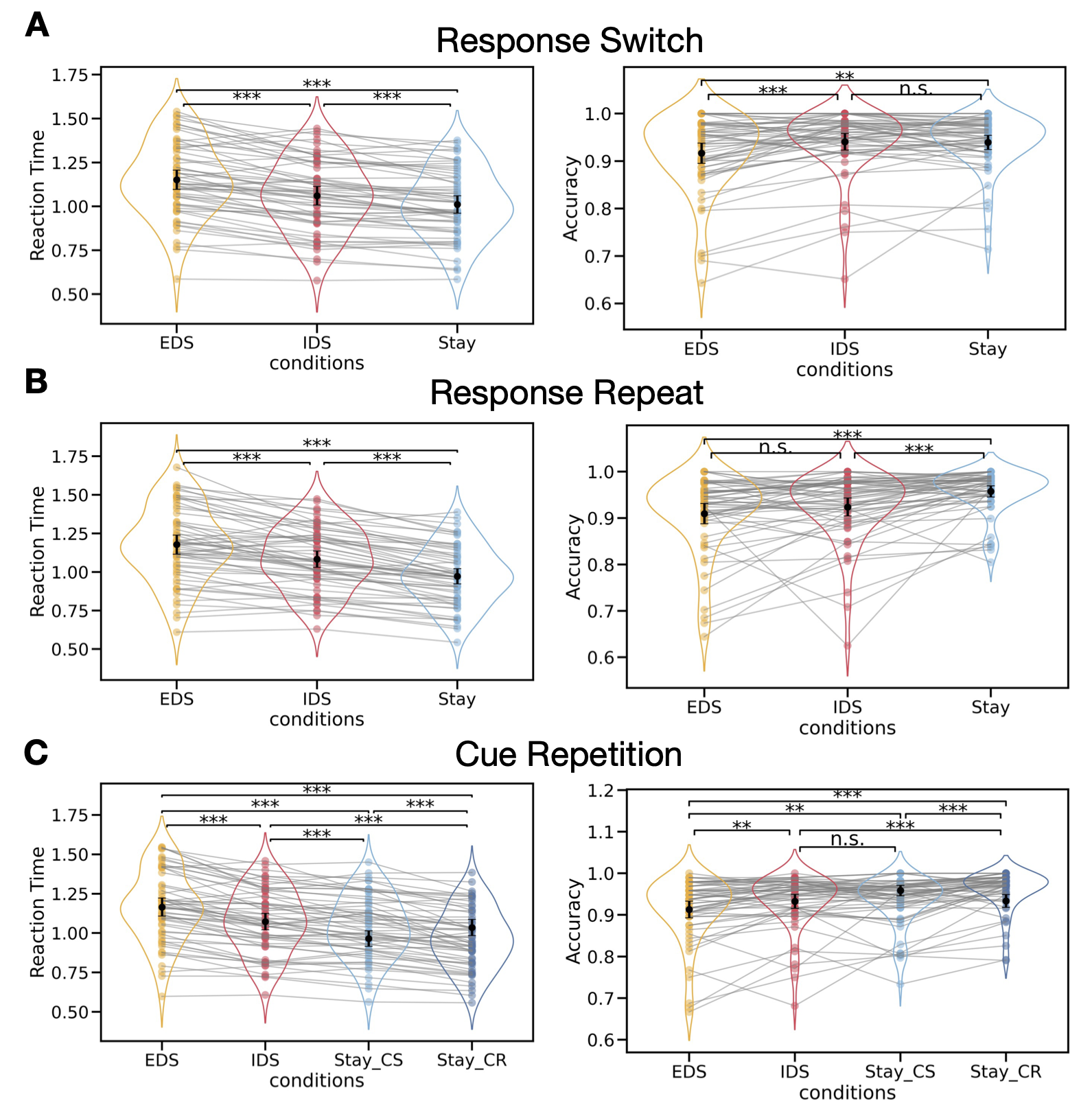

Supplement: S1 Fig — (A) Behavioral results for response switching. (B) Behavioral results for response repetition. (C) Behavioral results for cue repetition. (A–C) The left panel is reaction time and the right panel is accuracy. *** p < 0.001; ** p < 0.01; n.s., nonsignificant. The error bar represents the 95% confidential interval. The black dot indicates the mean value, while the colored dot represents data from individual subjects. Lines connect the data points for each subject across different conditions. Data used for (A–C) can be found in S1 Data, specifically in the sheet labeled “S1A Fig,” “S1B Fig,” and “S1C Fig.” (TIFF) [file pbio.3002937.s001.tiff]

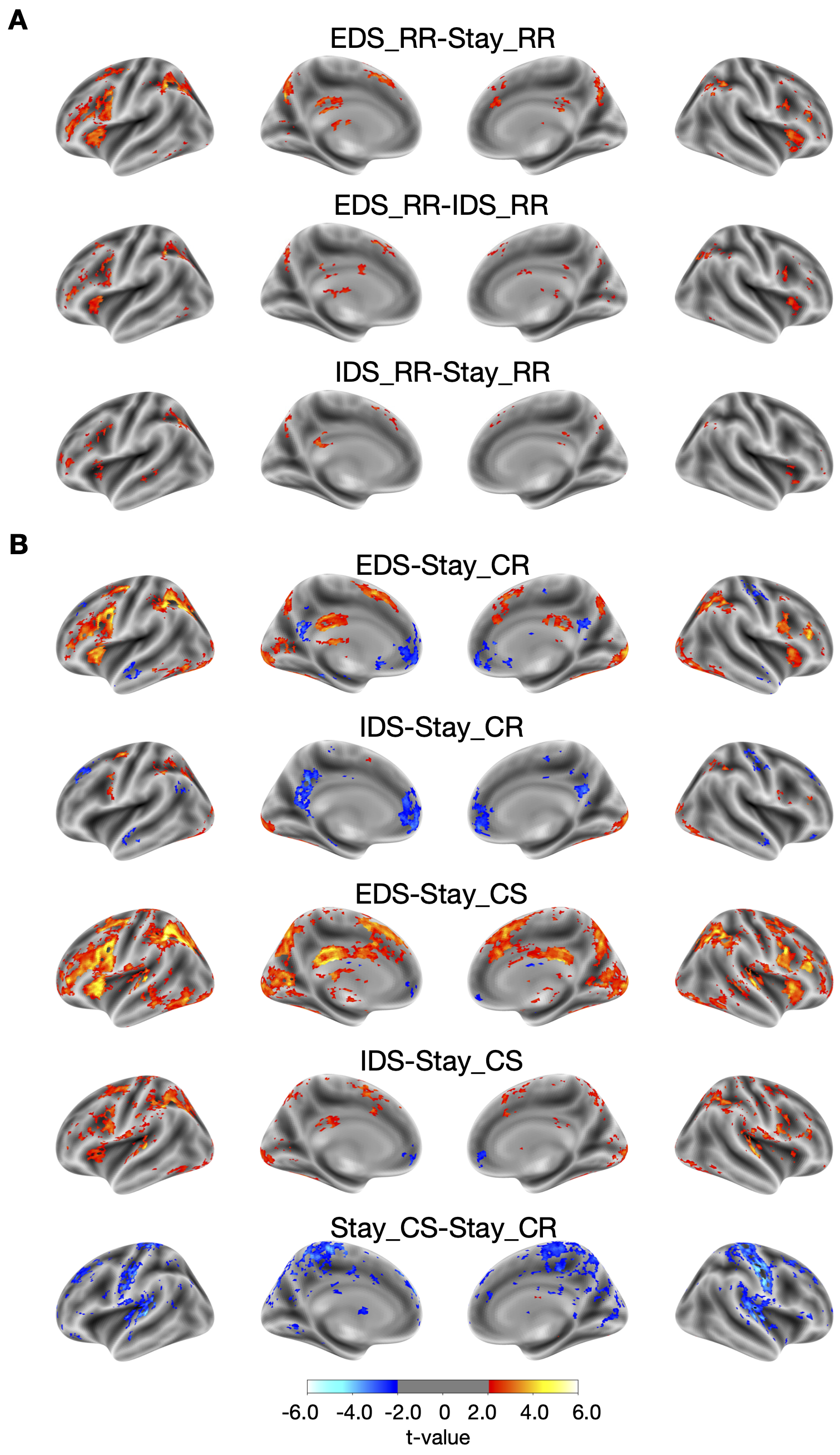

Supplement: S2 Fig — (A) Contrasts between hierarchical task-switching conditions, accounting for trials where the current trial’s decision (choosing “yes” or “no”) repeats from the previous trial (RR). (B) Contrasts between task-switching conditions, separate trials where the cue of the current trial either repeats (CR) or switches (CS) from the previous trial. The results were first thresholded at a voxel-level threshold of p < 0.05, followed by cluster correction procedure with a cluster level threshold of p < 0.05, only showing clusters with a minimum cluster size (k) of 58 voxels. Clusters were defined as groups of voxels that are connected by sharing a face with their neighboring voxels. The group statistical maps presented in S2 Fig can be accessed at https://identifiers.org/neurovault.collection:18728. (TIFF) [file pbio.3002937.s002.tiff]

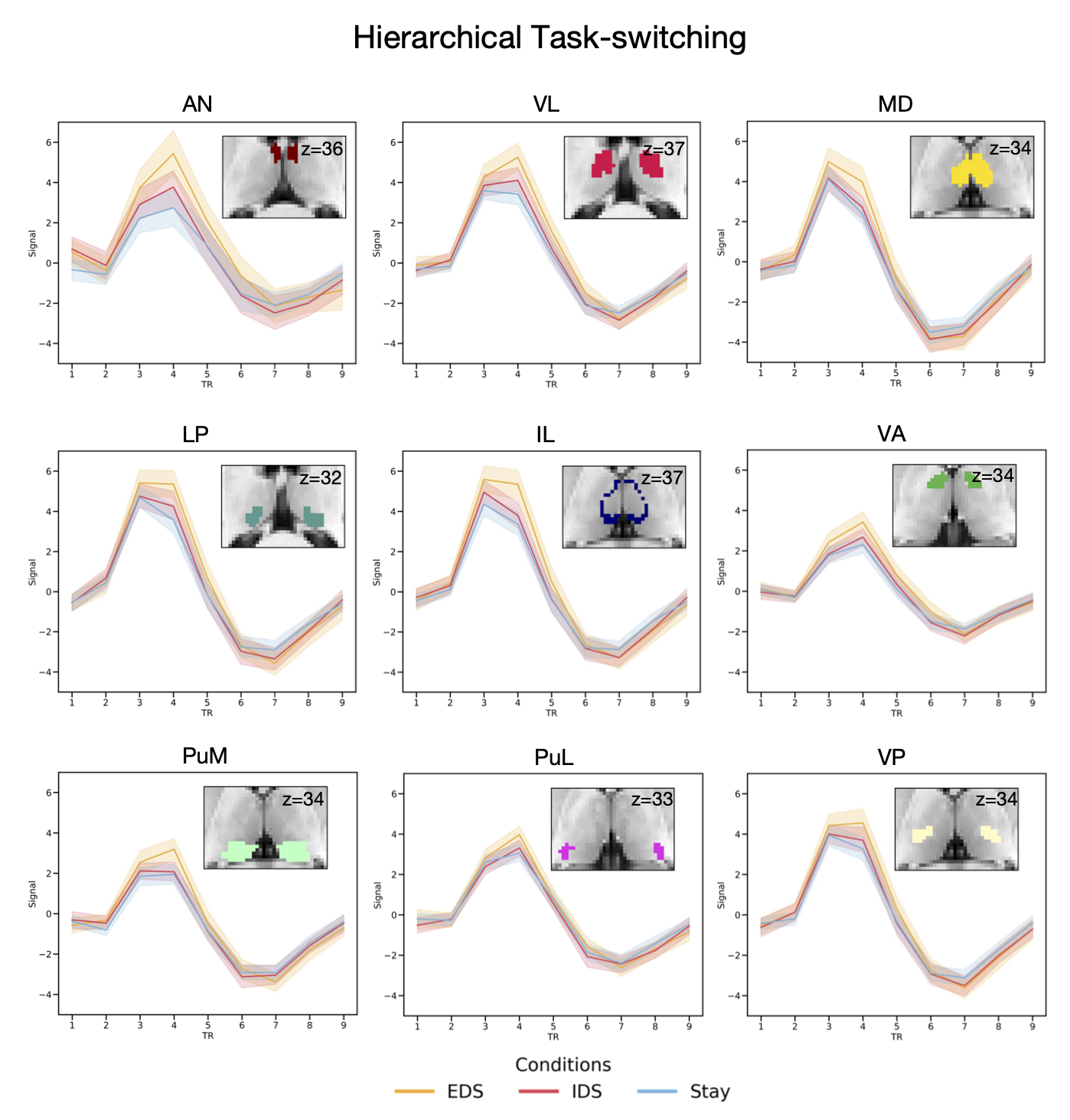

Supplement: S3 Fig — Data used for this figure can be found in S1 Data, specifically in the sheet labeled “S3 Fig.” (TIFF) [file pbio.3002937.s003.tiff]

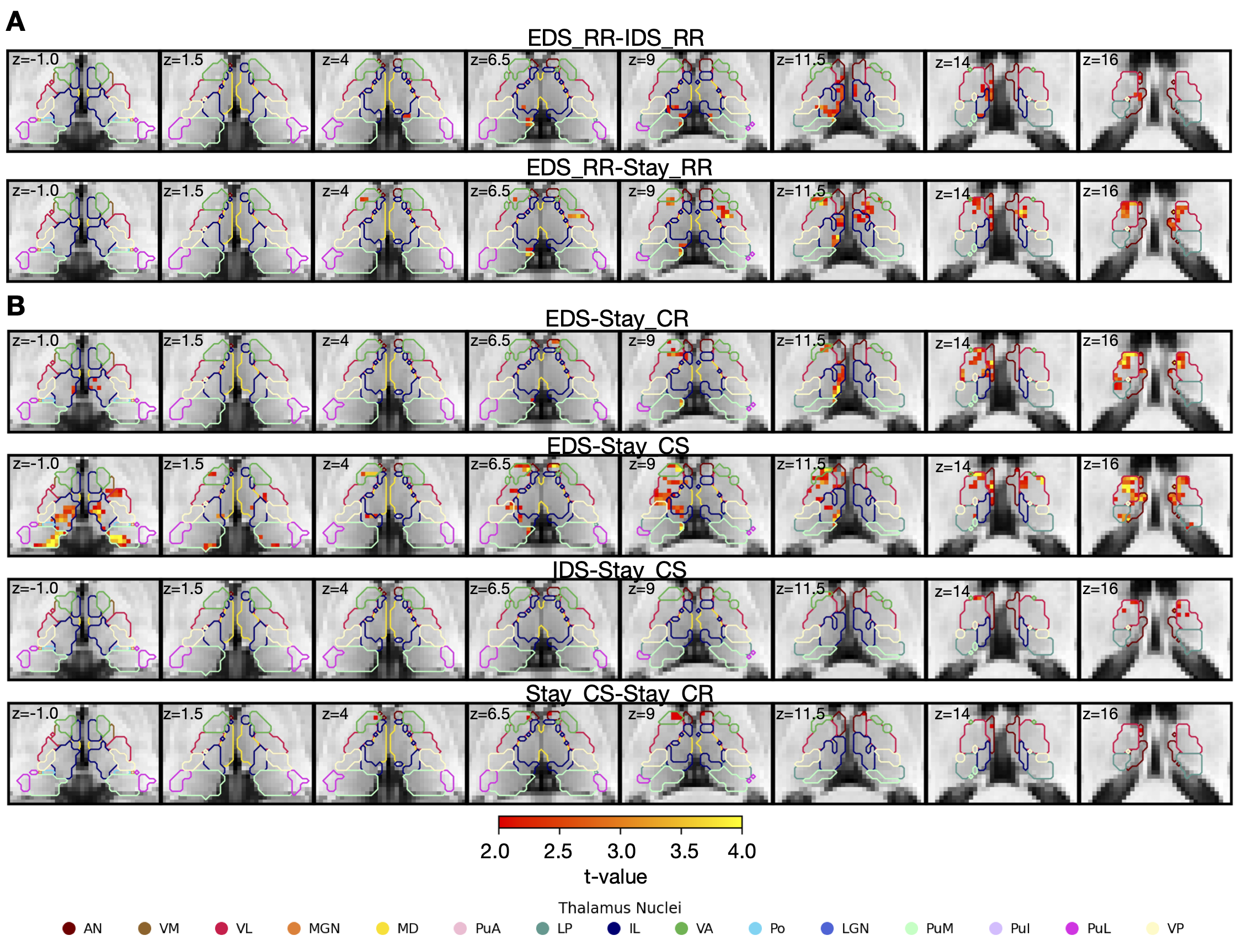

Supplement: S4 Fig — (A) Contrasts between conditions, trials where current trial’s decision (choosing “yes” or “no”) repeats the previous trial (RR). (B) Contrasts between task-switching conditions, separate trials where the cue of the current trial either repeats (CR) or switches (CS) from the previous trial. The results were first thresholded at a voxel-level threshold of p < 0.05, followed by cluster correction procedure with a cluster level threshold of p < 0.05, only showing clusters with a minimum cluster size (k) of 58 voxels. Clusters were defined as groups of voxels that are connected by sharing a face with their neighboring voxels. The group statistical maps presented in S4 Fig can be accessed at https://identifiers.org/neurovault.collection:18728. (TIFF) [file pbio.3002937.s004.tiff]

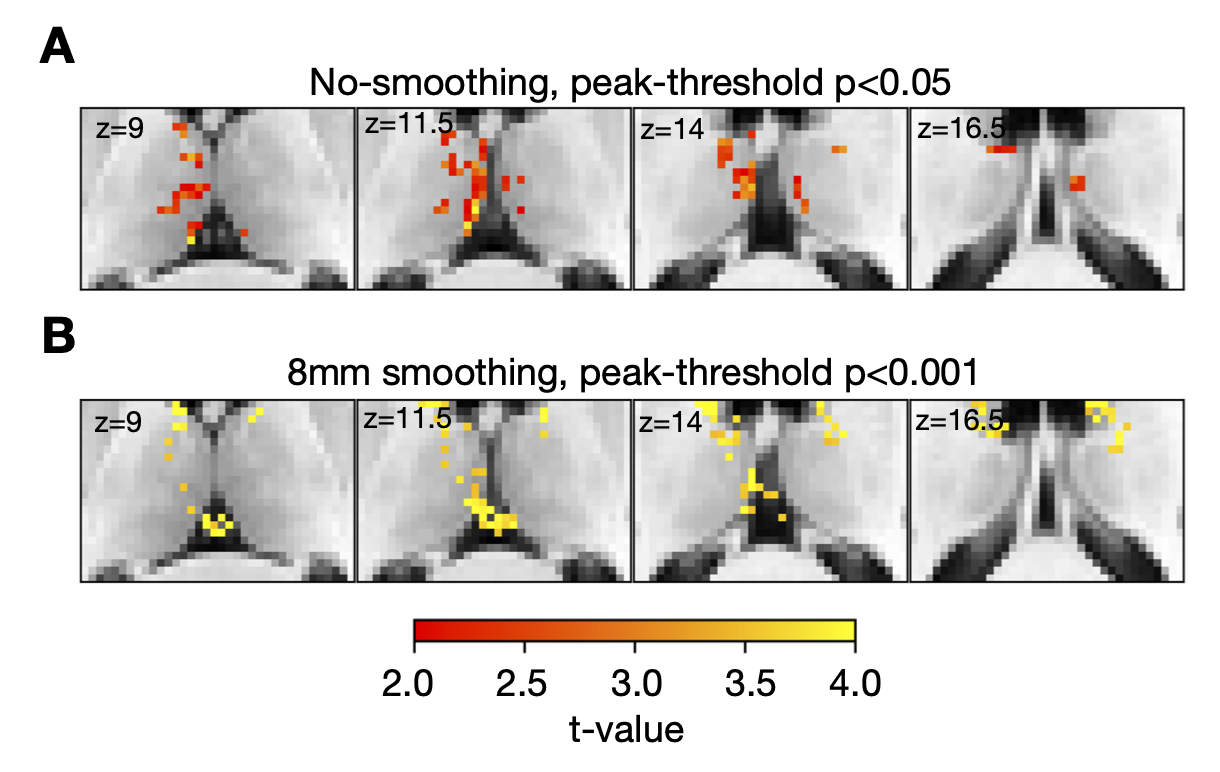

Supplement: S5 Fig — (A) Thalamic data without spatial smoothing in EDS-IDS condition, analyzed with a peak threshold of p < 0.05, followed by cluster corrected at p < 0.05 with a minimum cluster size of 58 voxels. (B) Thalamic data smoothed with an 8 mm kernel in EDS-IDS condition, using a peak threshold of p < 0.001, followed by cluster correction at p < 0.05 with a minimum cluster size of 42 voxels. Clusters were defined by whether adjacent voxels touch either in-plane or at points. The group statistical maps presented in S5 Fig can be accessed at https://identifiers.org/neurovault.collection:18728. (TIFF) [file pbio.3002937.s005.tiff]

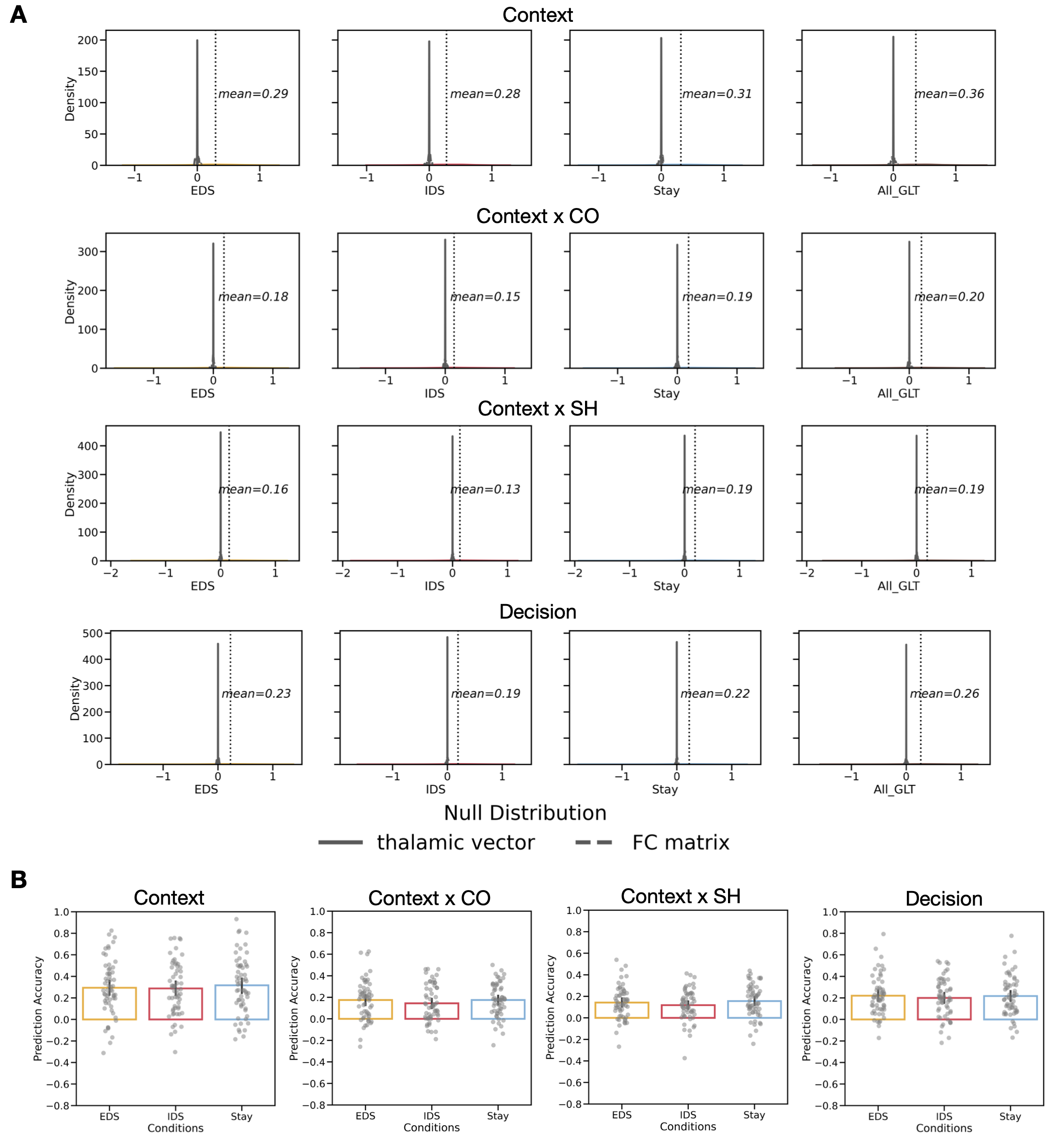

Supplement: S6 Fig — (A) Thalamocortical interaction model compared to null models. (B) Model performance of 3 hierarchical task switching conditions when predicting different cortical representations. Data used for (A) and (B) can be found in S1 Data, specifically in the sheet labeled “S6A Fig” and “S6B Fig.” (TIFF) [file pbio.3002937.s006.tiff]

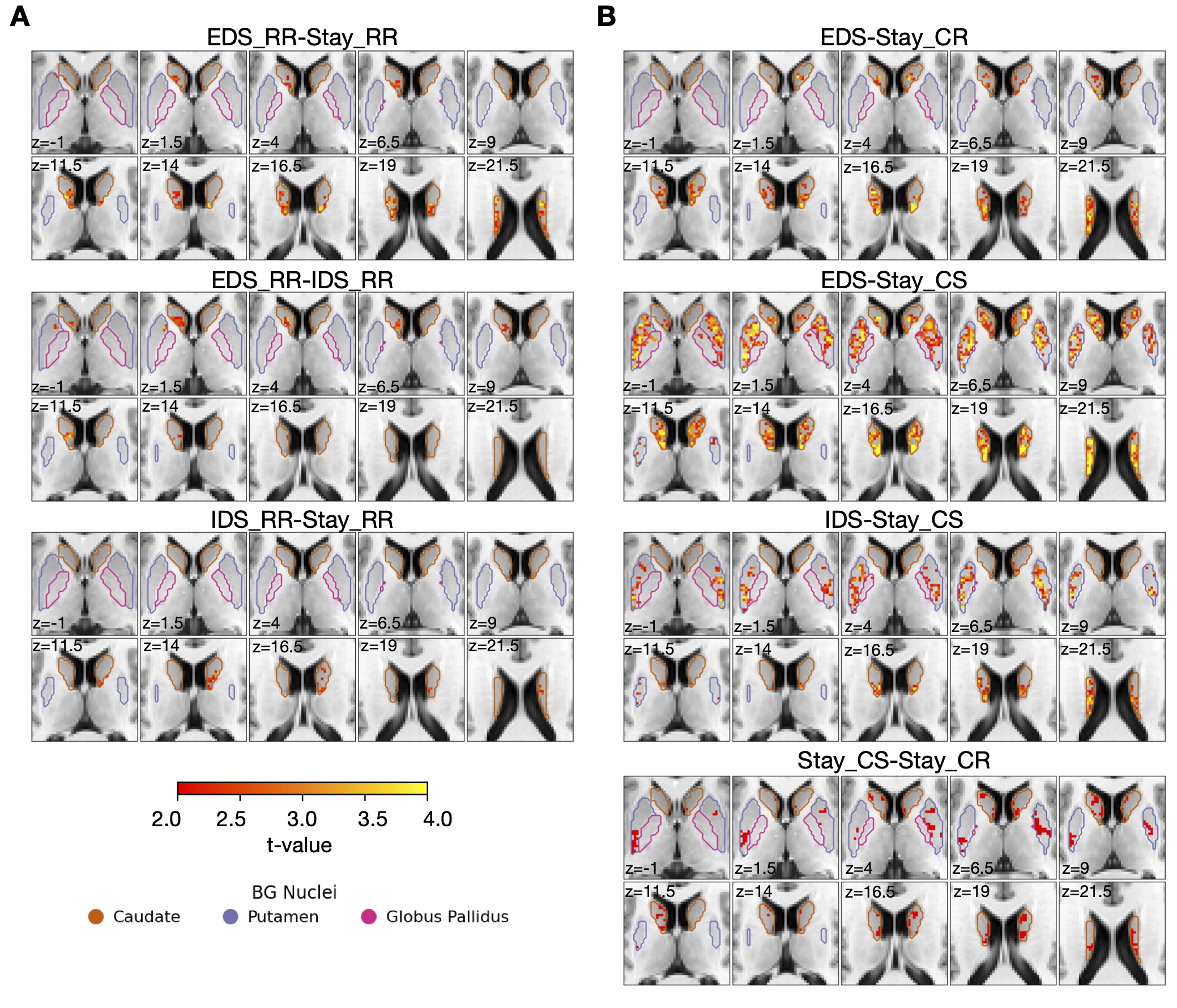

Supplement: S7 Fig — (A) Contrasts between conditions, separating trials where current trial’s decision (choosing “yes” or “no”) repeats from the previous trial (RR). (B) Contrasts between task-switching conditions, where the cue in the current trial either repeats (CR) or switches (CS) from the previous trial. The results were first thresholded at a voxel-level threshold of p < 0.05, followed by cluster correction procedure with a cluster level threshold of p < 0.05, only showing clusters with a minimum cluster size (k) of 58 voxels. Clusters were defined as groups of voxels that are connected by sharing a face with their neighboring voxels. The group statistical maps presented in S7 Fig can be accessed at https://identifiers.org/neurovault.collection:18728. (TIFF) [file pbio.3002937.s007.tiff]

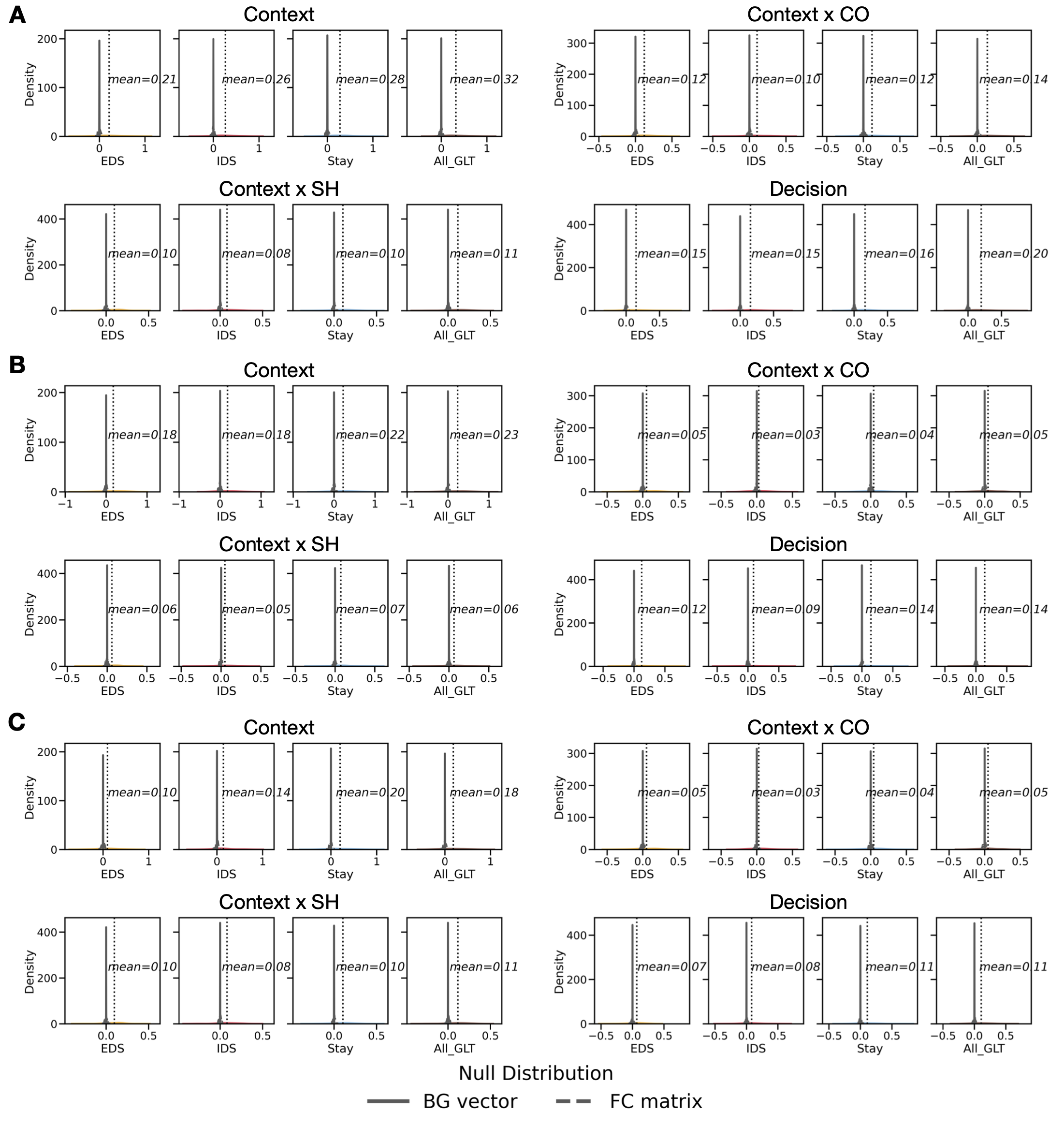

Supplement: S8 Fig — (A) Caudate-cortical interaction model compared to 2 null models. (B) Putamen-cortical interaction model compared to 2 null models. (C) Globus pallidus-cortical interaction model compared to 2 null models. Data used for this figure can be found in S1 Data, specifically in the sheet labeled “S8 Fig.” (TIFF) [file pbio.3002937.s008.tiff]

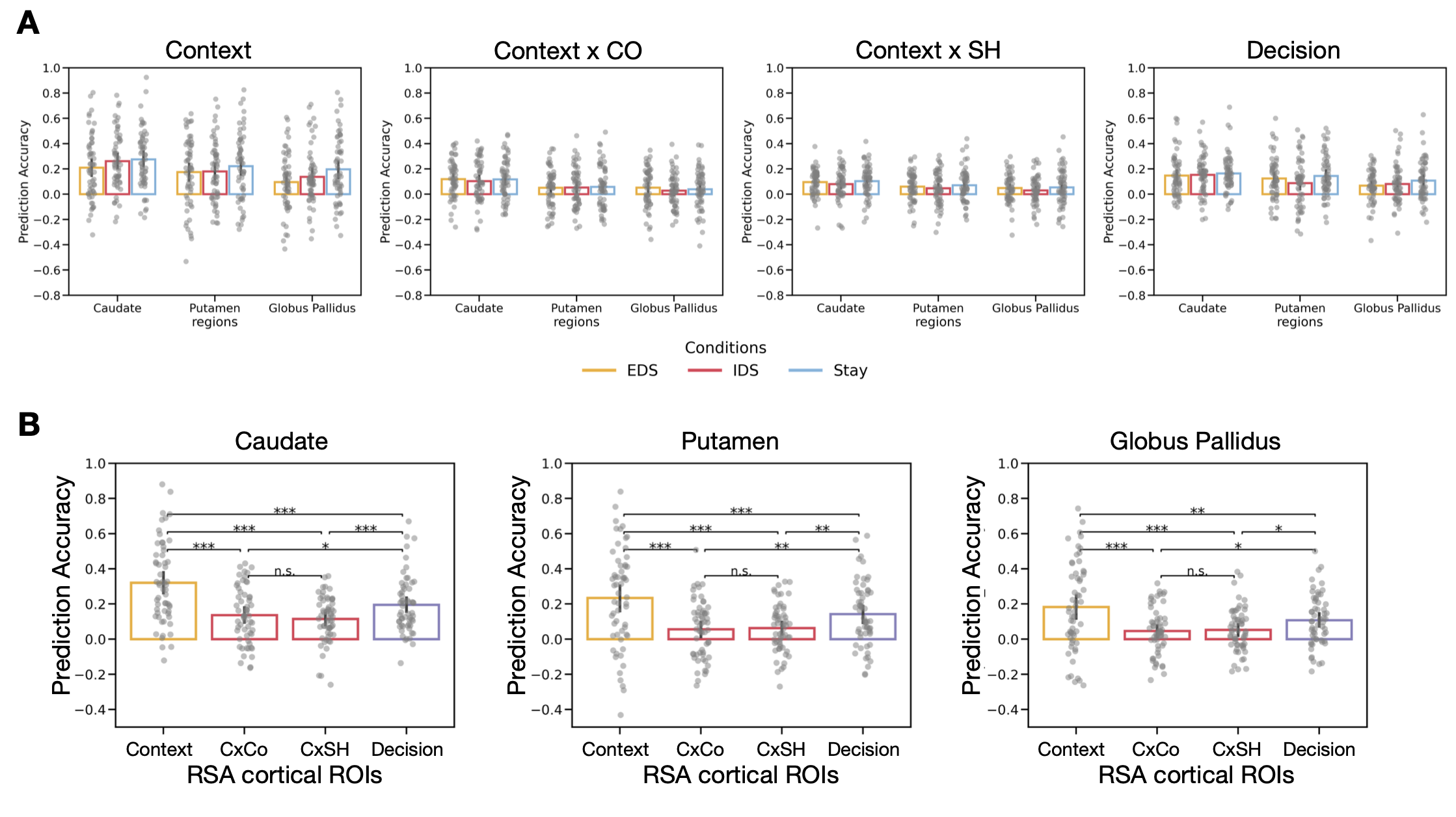

Supplement: S9 Fig — (A) Model performance of 3 hierarchical task switching conditions when predicting different cortical representations. (B) Model performance of predicting different cortical representations. *p < 0.05; **p < 0.01; **p < 0.001; n.s., not significant. Data used for this figure can be found in S1 Data, specifically in the sheet labeled “S9 Fig.” (TIFF) [file pbio.3002937.s009.tiff]
