# Supplementary material for: The thalamus encodes and updates context representations during hierarchical cognitive control
Source: PLoS Biol. 2024 Dec 2;22(12):e3002937. doi: 10.1371/journal.pbio.3002937 (PMC11637348; doi:10.1371/journal.pbio.3002937)
Supplement: S1 Text — (DOCX) [file pbio.3002937.s010.docx]

**Supporting information**

**S1 Text. Brain regions activated during hierarchical task-switching**

**Table A: Brain regions activated during EDS**

| Voxels | CM coordinates | Regions | t-value (mean ± SEM) |
| --- | --- | --- | --- |
| 35996 | [-0.5, 65.8, -0.7] | intraparietal sulcus; lateral occipital gyrus;  the fusiform gyrus; the inferior temporal gyrus;  thalamus; basal ganglia | 3.72 ± 0.01 |
| 2838 | [44.6, -14.8, 32.1] | left middle frontal gyrus | 3.25 ± 0.02 |
| 1498 | [1.1, -12.2, 50.5] | dorsal medial prefrontal cortex | 3.33 ± 0.03 |
| 963 | [-47.4, -35.3, 25.2] | right superior frontal gyrus | 3.21 ± 0.03 |
| 487 | [36.7, -18.7, 3.9] | left insula | 3.58 ± 0.06 |
| 434 | [-45.5, -8.7, 28.7] | right middle frontal gyrus | 3.17 ± 0.04 |
| 433 | [-36.2, -21.5, 2.5] | right insula | 3.42 ± 0.04 |
| 273 | [-0.2, 30.6, 27.6] | posterior cingulate cortex | 3.23 ± 0.05 |
| 229 | [-37.4, -0.9, 56.3] | right precentral sulcus | 2.92 ± 0.05 |
| 186 | [0.9, -5.9, 83.0] | left superior frontal gyrus | 2.90 ± 0.05 |
| 154 | [11.7, 74.7, 10.4] | left cuneus | 2.77 ± 0.05 |
| 132 | [44.1, 3.0, 10.8] | left precentral sulcus | 2.97 ± 0.07 |
| 101 | [23.6, 51.4, 24.6] | left precuneus | 2.75 ± 0.06 |
| 101 | [60.4, 20.6, 26.9] | left postcentral sulcus | 2.65 ± 0.05 |

**Table B: Brain regions activated during IDS**

| Voxels | CM coordinates | Regions | t-value (mean ± SEM) |
| --- | --- | --- | --- |
| 28981 | [-1.4, 67.5, -0.7] | intraparietal sulcus; lateral occipital gyrus; the fusiform gyrus; the inferior temporal gyrus; thalamus; basal ganglia | 3.67 ± 0.01 |
| 1277 | [47.8, -13.6, 30.4] | left middle frontal gyrus | 3.13 ± 0.03 |
| 964 | [2.2, -9.8, 51.4] | dorsal medial prefrontal cortex | 3.14 ± 0.03 |
| 502 | [-47, -31.7, 23.7] | right superior frontal gyrus | 3.20 ± 0.04 |
| 323 | [30.4, 5.3, 53.5] | left precentral sulcus | 3.17 ± 0.05 |
| 286 | [35.6, -18.6, 6.6] | left insula | 3.22 ± 0.05 |
| 278 | [-45.0, -8.0, 28.4] | right middle frontal gyrus | 3.26 ± 0.06 |
| 249 | [-35.8, -21.5, 4.4] | right insula | 2.93 ± 0.04 |
| 174 | [59.4, 20.0, 25.7] | left postcentral sulcus | 2.67 ± 0.04 |
| 163 | [3.3, -14.8, 79.6] | left superior frontal gyrus | 2.84 ± 0.05 |
| 155 | [44.1, 2.9, 13.1] | left precentral sulcus | 3.16 ± 0.08 |
| 101 | [-37.0, 0.4, 56.0] | right precntral sulcus | 2.84 ± 0.07 |
| 68 | [-45.5, -50.3, 23.1] | right superior frontal gyrus | 2.89 ± 0.10 |
| 60 | [39.4, 23.3, 57.2] | left precentral sulcus | 2.94 ± 0.10 |

**Table C: Brain regions activated during Stay**

| Voxels | CM coordinates | Regions | t-value (mean ± SEM) |
| --- | --- | --- | --- |
| 26416 | [-1.6, 67.0, -1.9] | intraparietal sulcus; lateral occipital gyrus; the fusiform gyrus; the inferior temporal gyrus; thalamus; basal ganglia | 3.71 ± 0.01 |
| 862 | [2.6, -9.7, 51.7] | dorsal medial prefrontal cortex | 3.07 ± 0.03 |
| 807 | [46.3, -10.6, 30.3] | left middle frontal gyrus | 3.07 ± 0.03 |
| 380 | [-46.5, -31.1, 23.3] | right superior frontal gyrus | 3.22 ± 0.05 |
| 251 | [35.1, -18.3, 7.9] | left insula | 3.15 ± 0.06 |
| 251 | [-44.3, -7.9, 28.3] | right superior frontal gyrus | 2.87 ± 0.04 |
| 224 | [-34.7, -21.6, 6.1] | right insula | 2.89 ± 0.05 |
| 195 | [44.1, 2.4, 13.2] | left insula | 3.36 ± 0.08 |
| 181 | [31.5, 5.6, 52.3] | left precentral sulcus | 2.93 ± 0.05 |
| 141 | [59.0, 20.0, 25.3] | left postcentral sulcus | 2.58 ± 0.04 |
| 104 | [1.4, -12.2, 80.8] | left superior frontal gyrus | 2.84 ± 0.06 |
| 78 | [-36.9, 0.6, 55.0] | right precentral sulcus | 2.79 ± 0.07 |
| 60 | [39.4, 20.7, 57.5] | left precentral sulcus | 2.90 ± 0.10 |

**Table D: Brain regions activated during EDS-Stay**

| Voxels | CM coordinates | Regions | t-value (mean ± SEM) |
| --- | --- | --- | --- |
| 46163 | [5.0, 29.5, 17.5] | superior and middle frontal gyrus; insula; posterior cingulate gyrus; cuneus; intraparietal sulcus; left thalamus; left basal ganglia | 3.05 ± 0.004 |
| 536 | [-12.3, 1.3, 14.7] | right thalamus; right basal ganglia | 2.89 ± 0.03 |
| 244 | [53.7, 20.5, 11.2] | left superior temporal gyrus | 2.73 ± 0.04 |
| 203 | [-55.8, 13.9, 7.4] | right superior temporal gyrus | 2.85 ± 0.05 |
| 163 | [-50.7, 30.9, -2.6] | right superior temporal sulcus | 2.64 ± 0.04 |
| 101 | [21.4, 27.5, 74.8] | left precentral sulcus | 2.70 ± 0.06 |

**Table E: Brain regions activated during EDS-IDS**

| Voxels | CM coordinates | Regions | t-value (mean ± SEM) | |
| --- | --- | --- | --- | --- |
| 5566 | [6.8, 62.8, 47.8] | intraparietal sulcus | 2.88 ± 0.01 | |
| 3948 | [42.7, -25.6, 22.6] | left middle frontal gyrus; left superior frontal gyrus; left insula | 2.87 ± 0.01 | |
| 1832 | [-43.6, -33.6, 22.4] | right middle frontal gyrus; right superior frontal gyrus; right insula | 2.78 ± 0.01 | |
| 1790 | [0.3, -20.7, 51.8] | dorsal medial prefrontal cortex | 2.80 ± 0.01 | |
| 613 | [0, 25.2, 31.6] | posterior cingulate cortex | 2.85 ± 0.03 | |
| 496 | [7.2, 7.6, 11.0] | thalamus; left basal ganglia | 2.66 ± 0.02 | |
| 493 | [-2.6, 74.8, 8.2] | cuneus | 2.67 ± 0.02 | |
| 448 | [51.9, 68.0, -21.5] | inferior temporal gyrus | 2.71 ± 0.03 |  |
| 437 | [-40.2, -20.2, 0.3] | right insula | 2.74 ± 0.03 |  |
| 273 | [62.2, 36.4, -5.6] | left middle temporal gyrus | 2.59 ± 0.03 |  |
| 263 | [-14.5, -5.4, 11.2] | right basal ganglia | 2.67± 0.03 |  |
| 5566 | [6.8, 62.8, 47.8] | intraparietal sulcus | 2.88 ± 0.01 |  |
| 3948 | [42.7, -25.6, 22.6] | left middle frontal gyrus; left superior frontal gyrus; left insula | 2.87 ± 0.01 |  |

**Table F: Brain regions activated during IDS-Stay**

| Voxels | CM coordinates | Regions | t-value (mean ± SEM) |
| --- | --- | --- | --- |
| 2526 | [20.6, 62.6, 56.7] | left intraparietal sulcus | 2.71 ± 0.01 |
| 735 | [52.2, -17.9, 30.5] | left caudal middle frontal sulcus | 2.68 ± 0.02 |
| 523 | [28.8, 3.4, 62.6] | left precentral sulcus | 2.80 ± 0.03 |
| 270 | [-55.5, 13.8, 7.7] | right superior temporal sulcus | 2.71 ± 0.03 |
| 252 | [10.9, -11.3, 74.7] | left caudal superior frontal sulcus | 2.72 ± 0.04 |
| 200 | [2.6, -9.4, 52.8] | left dorsal medial prefrontal cortex | 2.64 ± 0.03 |
| 177 | [56.5, 21.0, 11.3] | left superior temporal sulcus | 2.61 ± 0.04 |
| 164 | [30.3, 40.3, 73.4] | left postcentral sulcus | 2.75 ± 0.05 |
| 153 | [-29.9, 2.0, 60.7] | right caudal middle frontal sulcus | 2.77 ± 0.05 |
| 114 | [6.0, 21.9, 86.2] | left precentral sulcus | 2.87 ± 0.06 |
| 96 | [54.0, 69.2, -13.8] | left inferior temporal gyrus | 2.89 ± 0.07 |
| 93 | [-48..7, -16.2, -4,1] | right insula | 2.69 ± 0.08 |
| 91 | [-60.2, 24.8, -38.9] | right inferior temporal gyrus | 2.97 ± 0.07 |
| 74 | [-61.3, 61.8, -11.5] | right inferior temporal gyrus | 2.69 ± 0.08 |
| 60 | [-37.0, 44.2, 39.9] | right intraparietal sulcus | 2.60 ± 0.07 |

**Table G: Brain regions activated during EDS_RR-Stay_RR**

| Voxels | CM coordinates | Regions | t-value (mean ± SEM) |
| --- | --- | --- | --- |
| 5821 | [34.6, 63.7, 54.0] | intraparietal sulcus | 2.86 ± 0.01 |
| 3331 | [44.9, -37.7, 14.0] | left middle frontal gyrus | 2.91 ± 0.01 |
| 1148 | [2.0, 1.9, 64.0] | dorsal medial prefrontal cortex | 2.81 ± 0.02 |
| 710 | [-49.6, -46.3, 1.5] | right middle frontal gyrus | 2.73 ± 0.02 |
| 578 | [48.4, -15.3, -6] | left insula | 2.91 ± 0.03 |
| 419 | [-34.1, -18.8, 6.5] | right insula | 2.75 ± 0.03 |
| 256 | [-32.4, -1.6, 66.5] | right caudal middle frontal gyrus | 2.78 ± 0.04 |
| 233 | [2.0, 34.5, 26.5] | posterior cingulate cortex | 2.88 ± 0.05 |
| 197 | [-13.5, 0.1, 21.5] | right thalamus, right basal ganglia | 2.64 ± 0.04 |
| 167 | [19.1, 22.5, 24.0] | left thalamus, left basal ganglia | 2.64 ± 0.04 |
| 145 | [-10.1, -29.1, 29.0] | right anterior cingulate cortex | 2.59 ± 0.04 |
| 131 | [57.0, 24.2, -43.5] | left inferior temporal gyrus | 2.98 ± 0.06 |
| 94 | [14.0, -5.0, 11.5] | left basal ganglia | 2.57 ± 0.06 |
| 93 | [3.7, 20.8, 11.5] | medial thalamus | 2.67 ± 0.05 |

**Table H: Brain regions activated during EDS_RR-IDS_RR**

| Voxels | CM coordinates | Regions | t-value (mean ± SEM) |
| --- | --- | --- | --- |
| 1342 | [38.0, 58.6, 49.0] | left intraparietal sulcus | 2.78 ± 0.02 |
| 1081 | [58.7, -18.8, 31.5] | left caudal middle frontal sulcus | 2.72 ± 0.02 |
| 1067 | [-8.4, 75.8, 69.0] | precuneus | 2.76 ± 0.02 |
| 779 | [39.8, -44.6, 4.0] | left rostral middle frontal sulcus | 2.71 ± 0.02 |
| 777 | [-37.6, 67.2, 66.5] | right intraparietal sulcus | 2.72 ± 0.02 |
| 604 | [3.7, -15.3, 51.5] | dorsal medial prefrontal cortex | 2.66 ± 0.02 |
| 259 | [-44.5, -34.2, 39.0] | right middle frontal gyrus | 2.62 ± 0.03 |
| 254 | [34.6, -20.5, 1.5] | left insula | 2.81 ± 0.04 |
| 205 | [29.5, -13.6, 61.5] | left superior frontal gyrus | 2.68 ± 0.04 |
| 198 | [2.0, 36.2, 34.0] | posterior cingulate cortex | 2.67 ± 0.04 |
| 197 | [-51.3, -10.2, 26.5] | right precentral sulcus | 2.63 ± 0.04 |
| 167 | [-49.6, -29.1, 21.5] | right rostral middle frontal sulcus | 2.71 ± 0.04 |
| 166 | [2.0, 34.5, 6.5] | medial thalamus | 2.60 ± 0.04 |
| 162 | [-34.1, -22.2, 9.0] | right insula | 2.58 ± 0.03 |
| 142 | [5.4, -3.3, 79.0] | left superior frontal gyrus | 2.79 ± 0.05 |

**Table I: Brain regions activated during IDS_RR-Stay_RR**

| Voxels | CM coordinates | Regions | t-value (mean ± SEM) |
| --- | --- | --- | --- |
| 1494 | [36.3, 70.6, 54.0] | left intraparietal sulcus | 2.73 ± 0.02 |
| 600 | [0.2, -29.1, 36.5] | dorsal medial prefrontal cortex | 2.58 ± 0.02 |
| 435 | [36.3, -49.7, 4.0] | left rostral middle frontal sulcus | 2.64 ± 0.03 |
| 411 | [57.0, -18.8, 31.5] | left caudal middle frontal sulcus | 2.68 ± 0.03 |
| 365 | [-35.9, 67.2, 46.5] | right intraparietal sulcus | 2.61 ± 0.03 |
| 299 | [-13.5, 101.6, -13.5] | right occipital cortex | 2.82 ± 0.03 |
| 217 | [44.9, -17.1, 6.5] | left insula | 2.73 ± 0.04 |
| 147 | [-35.9, -22.2, 1.5] | right insula | 2.57 ± 0.03 |
| 119 | [-11.8, 70.6, 39.0] | right parieto-occipital sulcus | 2.60 ± 0.05 |
| 116 | [31.2, 3.6, 64.0] | left precentral sulcus | 2.73 ± 0.05 |
| 106 | [-44.5, 43.1, 46.5] | right intraparietal sulcus | 2.58 ± 0.04 |
| 103 | [48.4, 39.7, -1.0] | left middle temporal sulcus | 2.53 ± 0.04 |
| 102 | [3.7, 41.4, 24.0] | posterior cingulate cortex | 2.67 ± 0.06 |
| 96 | [-49.6, -30.8, 34.0] | right rostral middle frontal gyrus | 2.61 ± 0.05 |
| 80 | [39.8, -30.8, 19] | left middle frontal gyrus | 2.64 ± 0.05 |

**Table J: Brain regions activated during EDS-Stay_CR**

| Voxels | CM coordinates | Regions | t-value (mean ± SEM) |
| --- | --- | --- | --- |
| 7339 | [32.9, 68.9, 46.5] | intraparietal sulcus | 3.07 ± 0.01 |
| 6763 | [41.5, 0.1, 36.5] | left middle frontal sulcus | 3.03 ± 0.01 |
| 1997 | [-53.1, -11.9, 26.5] | right middle frontal sulcus | 2.91 ± 0.02 |
| 393 | [7.1, 72.3, 11.5] | occipital cortex | 2.70 ± 0.03 |
| 379 | [2.0, 31.1, 29.0] | posterior cingulate cortex | 3.06 ± 0.04 |
| 378 | [8.8, 3.6, 14.0] | left thalamus; left basal ganglia | 2.83 ± 0.04 |
| 359 | [-46.2, -17.1, -6.0] | right insula | 2.87 ± 0.04 |
| 266 | [-11.8, 1.9, 16.5] | right thalamus; right basal ganglia | 2.78 ± 0.04 |
| 148 | [-44.5, -53.2, 6.5] | right rostral middle frontal sulcus | 2.61 ± 0.04 |

**Table K: Brain regions activated during IDS-Stay_CR**

| Voxels | CM coordinates | Regions | t-value (mean ± SEM) |
| --- | --- | --- | --- |
| 1413 | [32.9, 68.9, 46.5] | left intraparietal sulcus | 2.83 ± 0.02 |
| 648 | [51.8, -8.5, 31.5] | left middle frontal sulcus | 2.73 ± 0.02 |
| 363 | [32.9, 5.3, 61.5] | left precentral sulcus | 2.80 ± 0.03 |
| 335 | [-35.9, 70.6, 64.0] | right intraparietal sulcus | 2.65 ± 0.03 |
| 260 | [0.2, -11.9, 49.0] | dorsal medial frontal cortex | 2.61 ± 0.03 |
| 174 | [29.5, -65.2, 26.5] | left rostral middle sulcus | 2.78 ± 0.05 |
| 154 | [-54.8, -11.9, 26.5] | right precentral sulcus | 2.72 ± 0.05 |
| 123 | [-46.2, 44.8, 61.5] | right intraparietal sulcus | 2.65 ± 0.05 |
| 105 | [53.5, -36.0, 24.0] | left superior frontal sulcus | 2.69 ± 0.06 |
| 87 | [-37.6, -1.6, 56.5] | right caudal middle frontal sulcus | 2.81 ± 0.06 |
| 76 | [-46.2, -32.5, 31.5] | right middle frontal sulcus | 2.88 ± 0.08 |
| 71 | [26.0, 41.4, 76.5] | left postcentral sulcus | 2.71 ± 0.06 |
