# Supplementary material for: The thalamus encodes and updates context representations during hierarchical cognitive control
Source: PLoS Biol. 2024 Dec 2;22(12):e3002937. doi: 10.1371/journal.pbio.3002937 (PMC11637348; doi:10.1371/journal.pbio.3002937)
Supplement: S2 Text — (DOCX) [file pbio.3002937.s011.docx]

**Supporting information**

**S2 Text. Noise ceiling metric**

For the voxel-to-parcellation thalamocortical interaction model, we used a one-way repeated measure (rm) ANOVA to compare the split-half noise ceiling of voxel-to-parcellation thalamocortical interaction model for each task-switching condition (EDS, IDS and Stay; Table A; Data used for this table can be found in S1 Data (S1_Data.xlsx), specifically in the sheet labeled “S2 Text”.).

**Table A: Noise ceiling results for voxel-to-parcellation thalamocortical interaction model**

| F-value  (2, 116) | p-value | EDS | IDS | Stay |
| --- | --- | --- | --- | --- |
|  |  | (mean ± SD) | (mean ± SD) | (mean ± SD) |
| 0.10 | 0.37 | 0.67 ± 0.07 | 0.66 ± 0.07 | 0.67 ± 0.07 |

For the voxel-to-voxel thalamocortical interaction model, we used a one-way rmANOVA to compare the different conditions (EDS, IDS, Stay and All) within each cortical representation regions separately (Table B).

**Table B: Noise ceiling results for voxel-to-voxel thalamocortical interaction model**

| Region | F-value  (3, 174) | p-value | EDS | IDS | Stay | All |
| --- | --- | --- | --- | --- | --- | --- |
|  |  |  | (mean ± SD) | (mean ± SD) | (mean ± SD) | (mean ± SD) |
| Context | 2.26 | 0.08 | 0.60 ± 0.04 | 0.60 ± 0.04 | 0.60 ± 0.04 | 0.61 ± 0.04 |
| Context x Color | 4.41 | 0.01 | 0.59 ± 0.03 | 0.59 ± 0.03 | 0.60 ± 0.03 | 0.60 ± 0.03 |
| Context x Shape | 4.00 | 0.01 | 0.59 ± 0.02 | 0.59 ± 0.02 | 0.59 ± 0.02 | 0.59 ± 0.02 |
| Decision | 1.24 | 0.3 | 0.59 ± 0.03 | 0.59 ± 0.02 | 0.59 ± 0.02 | 0.60 ± 0.02 |

For the voxel-to-voxel caudate-cortical interaction model, we used a one-way rmANOVA to compare the different conditions (EDS, IDS, Stay and All) within each cortical representation regions separately (Table C; Data used for this table can be found in S1 Data (S1_Data.xlsx), specifically in the sheet labeled “S2 Text”).

**Table C: Noise ceiling results for voxel-to-voxel caudate-cortical interaction model**

| Region | F-value  (3, 174) | p-value | EDS | IDS | Stay | All |
| --- | --- | --- | --- | --- | --- | --- |
|  |  |  | (mean ± SD) | (mean ± SD) | (mean ± SD) | (mean ± SD) |
| Context | 2.74 | 0.04 | 0.59 ± 0.03 | 0.60 ± 0.03 | 0.59 ± 0.03 | 0.60 ± 0.03 |
| Context x Color | 1.95 | 0.12 | 0.58 ± 0.02 | 0.59 ± 0.02 | 0.59 ± 0.02 | 0.59 ± 0.02 |
| Context x Shape | 2.50 | 0.06 | 0.58 ± 0.01 | 0.58 ± 0.01 | 0.58 ± 0.01 | 0.58 ± 0.01 |
| Decision | 2.28 | 0.08 | 0.58 ± 0.01 | 0.57 ± 0.02 | 0.59 ± 0.01 | 0.59 ± 0.02 |

For the voxel-to-voxel putamen-cortical interaction model, we used a one-way rmANOVA to compare the different conditions (EDS, IDS, Stay and All) within each cortical representation regions separately (Table D; Data used for this table can be found in S1 Data (S1_Data.xlsx), specifically in the sheet labeled “S2 Text”).

**Table D: Noise ceiling results for voxel-to-voxel putamen-cortical interaction model**

| Region | F-value  (3, 174) | p-value | EDS | IDS | Stay | All |
| --- | --- | --- | --- | --- | --- | --- |
|  |  |  | (mean ± SD) | (mean ± SD) | (mean ± SD) | (mean ± SD) |
| Context | 3.64 | 0.01 | 0.59 ± 0.03 | 0.59 ± 0.03 | 0.60 ± 0.04 | 0.60 ± 0.03 |
| Context x Color | 3.40 | 0.02 | 0.58 ± 0.02 | 0.58 ± 0.02 | 0.59 ± 0.02 | 0.59 ± 0.02 |
| Context x Shape | 4.15 | 0.01 | 0.58 ± 0.01 | 0.58 ± 0.02 | 0.59 ± 0.02 | 0.59 ± 0.02 |
| Decision | 3.06 | 0.03 | 0.59 ± 0.02 | 0.58 ± 0.02 | 0.59 ± 0.02 | 0.59 ± 0.02 |

For the voxel-to-voxel globus pallidus-cortical interaction model, we used a one-way rmANOVA to compare the different conditions (EDS, IDS, Stay and All) within each cortical representation regions separately (Table E; Data used for this table can be found in S1 Data (S1_Data.xlsx), specifically in the sheet labeled “S2 Text”).

**Table E: Noise ceiling results for voxel-to-voxel globus pallidus-cortical interaction model**

| Region | F-value  (3, 174) | p-value | EDS | IDS | Stay | All |
| --- | --- | --- | --- | --- | --- | --- |
|  |  |  | (mean ± SD) | (mean ± SD) | (mean ± SD) | (mean ± SD) |
| Context | 2.92 | 0.04 | 0.59 ± 0.03 | 0.59 ± 0.03 | 0.60 ± 0.04 | 0.59 ± 0.03 |
| Context x Color | 0.39 | 0.76 | 0.58 ± 0.02 | 0.58 ± 0.02 | 0.58 ± 0.02 | 0.58 ± 0.02 |
| Context x Shape | 0.28 | 0.84 | 0.58 ± 0.01 | 0.58 ± 0.02 | 0.58 ± 0.023 | 0.58 ± 0.02 |
| Decision | 0.70 | 0.56 | 0.58 ± 0.02 | 0.58 ± 0.02 | 0.59 ± 0.02 | 0.59 ± 0.02 |
