# Supplementary material for: The thalamus encodes and updates context representations during hierarchical cognitive control
Source: PLoS Biol. 2024 Dec 2;22(12):e3002937. doi: 10.1371/journal.pbio.3002937 (PMC11637348; doi:10.1371/journal.pbio.3002937)
Supplement: Attachment — Submitted filename: Response_to_Reviewers.pdf [file pbio.3002937.s013.pdf]

We thank the reviewers their thorough review and helpful comments. Below, we provide our responses to each comment and outline the corresponding revisions made to the manuscript. All changes in the manuscript are highlighted in red for easy reference.

REVIEWS:

Reviewer #1 (Burkhard Pleger): This fMRI study explores the role of thalamic representation and thalamocortical interactions in hierarchical cognitive control in humans. The findings reveal that specific thalamic nuclei, including the anterior, mediodorsal, ventrolateral, and pulvinar, exhibit stronger responses during task context switching. Decoding analysis indicates that thalamic activity preferentially encodes task contexts within hierarchical task structures. To further understand the contribution of thalamocortical interactions to these representations, the researchers developed a thalamocortical functional interaction model. This data-driven model outperformed other models, particularly in predicting cortical activity patterns associated with context representations.

These results highlight the crucial role of thalamic activity and thalamocortical interactions in facilitating contextually guided hierarchical cognitive control. The task is exceptionally well-designed to address the scientific question effectively. The study is novel and innovative, providing fresh insights into the underexplored role of the thalamus in cognitive processing. It significantly contributes to our understanding of how the thalamus supports complex cognitive functions through intricate brain network interactions.

I have only a few points I would like the authors to consider:

1. For MVPA, the authors conducted voxel-wise t-tests comparing the decoded results against a chance level of 0.5. In the results section, they report the highest decoding accuracy across almost all thalamic nuclei, including the anterior, ventroanterior, ventrolateral, mediodorsal, intralaminar, medial pulvinar, and lateral pulvinar. However, the mean accuracy for each nucleus was 0.52 ± 0.004. Despite significant t-tests this seems rather unconvincing to me.

Response: Thank you for your comment. When applying MVPA to fMRI data to decode abstract and control-related representations, decoding accuracy is often lower compared to other data sources (e.g., EEG, electrophysiology) and lower than when decoding visual stimuli in the occipital-temporal cortices. Typically, decoding accuracy is only a few percentage points above chance. This is a well-documented phenomenon discussed in several published reports (e.g., Bhandari et al., 2018). The difficulty in decoding control representations using fMRI remains unclear but may be due to factors such as

hemodynamic blurring, partial volume effects, or signals from mixed-selectivity neurons being blurred within voxels. In the case of the thalamus, which is located near noise sources and receives mixed, converging inputs from multiple cortical areas, we expect the decoding effect size to be even weaker than that observed in the cortex.

Given this consideration, it is essential that we report only robust and reliable decoding results. As suggested by Reviewer 2, we have implemented a cluster correction method based on the nonparametric cluster permutation procedure published by Stelzer et al. (2013). This method performs statistical inferences using empirically derived null distributions instead of using parametric distributions, ensuring that any biases are reflected in the null data, with each voxel having its own null distribution. Additionally, we adopted a more stringent threshold, applying a voxel-level threshold of $p < 0.001$ and a cluster-level threshold of $p < 0.001$. With this procedure, we are confident in the robustness of our results. Figure 4 has also been updated to reflect these changes.

Bhandari, A., Gagne, C., & Badre, D. (2018). Just above chance: is it harder to decode information from prefrontal cortex hemodynamic activity patterns?. *Journal of cognitive neuroscience*, *30*(10), 1473-1498.

Stelzer, J., Chen, Y., & Turner, R. (2013). Statistical inference and multiple testing correction in classification-based multi-voxel pattern analysis (MVPA): random permutations and cluster size control. *Neuroimage*, *65*, 69-82.

2. Why authors haven't applied the RSA approach to dissect the thalamic nuclei according to the different representations of the hierarchical task structures. The methods described for identifying cortical representations through ROI RSA to fit five representation similarity models and assess different representations are thorough and insightful. It would be beneficial to understand if the different thalamic nuclei also encode these hierarchical task representations similarly.

Response: Thank you for your comment. We conducted an RSA analysis on different thalamic nuclei to explore whether they encode distinct hierarchical task representations. This analysis showed that the mediodorsal (MD) and medial pulvinar (PuM) thalamic nuclei encoded relatively strong contextual representation (MD: t-value = 1.67, uncorrected p = 0.048; PuM: t-value = 1.68, uncorrected p = 0.047). We did not find any other significant effects. While these results are consistent with the MVPA decoding results described above, after correcting for multiple comparisons, these results were not statistically significant. Given the lack of statistically significant findings after correction, we have

decided not to incorporate these analyses in the current manuscript. We suspect that RSA, given it captures the similarity in high-dimensional voxel-wise response patterns among conditions, might work better with larger regions of interest (ROIs) that have more voxels. Given our focus on functional anatomy, we decided against applying RSA using the whole thalamus as a ROI. Furthermore, exploring these methodological differences is beyond the scope of this manuscript.

3. As the authors correctly pointed out, the basal ganglia (BG) are hypothesized to regulate thalamocortical circuits by inhibiting or disinhibiting them when cortical representations require updating. However, since the BG lack direct projections to the cortex, it is likely that the cortical representations of task information involve thalamocortical interactions modulated by the inhibitory output from the BG. Given this framework, authors could directly test their hypothesis. Network models, such as non-linear Dynamic Causal Modeling (https://www.ncbi.nlm.nih.gov/pmc/articles/PMC2636907/), offer the possibility to directly test the inhibitory/excitatory influence of BG time series on thalamocortical connections.

Response: We appreciate the reviewer's suggestion to use nonlinear Dynamic Causal Modeling (DCM) to directly test the inhibitory/excitatory influence of basal ganglia (BG) on thalamocortical interactions. We agree that such an approach could provide valuable insights into the regulatory role of the BG within thalamocortical circuits, especially in relation to task-related cortical representations. However, implementing nonlinear DCM in the context of our study would involve a high degree of complexity, as it requires modeling interactions between two subcortical structures (BG and thalamus) alongside multiple cortical ROIs that encode different hierarchical representations. The model space is considerable and computational rather intractable.

Given the methodological complexity and the extensive nature of nonlinear DCM, we believe it would be more appropriate to pursue this analysis in a future study that is specifically dedicated to exploring thalamocortical interactions modulated by the BG. This future work could focus more directly on the dynamics of these circuits, allowing for a thorough and systematic investigation of the BG's role in modulating task-related cortical representations via the thalamus. In the current manuscript, our primary goal is to explore thalamocortical interactions and hierarchical task representations using more straightforward approaches, while leaving more complex network modeling techniques for future, dedicated research.

Reviewer #2: Chen et al. describe an fMRI study of the contributions of the thalamus to hierarchical control. Some prominent models of these control mechanisms describe a PFC-basal ganglia-thalamic circuit that gates the flow of information to selectively activate representations based on hierarchically organized contingencies. As the authors point out, the role of the thalamus in this process has not been well-examined, with most models assuming its function to be equivalent to the parallel circuitry involved in controlling motor responses. Chen et al. demonstrate that parts of the thalamus respond to higher-order control demands and maintain a representation of the higher order contextual variable in a hierarchical control task. They also use 'activity flow mapping' to show that thalamic activity is predictive of cortical responses - mediated by functional connectivity between thalamic and cortical voxels. This study makes an important contribution to our understanding of control processes by providing a detailed examination of the thalamic circuitry that supports hierarchical control. The manuscript is well-written, clearly organized and timely. However, some important aspects of the methods and statistical choices were missing or not clear. I also have some suggestions and questions about analyses that I will describe below.

1. The authors convincingly show that task representations in the thalamus and cortex are related, but do not show any link between these representations and behavior. This might be somewhat out of the scope of this study, but it would be interesting to know if the thalamic context representation is correlated with task performance within-subject or across individuals?

Response: Thank you for your suggestion. As requested, we have conducted analysis of the correlation between thalamic decoding accuracy of contextual information and behavioral performance in EDS (response time and accuracy) across subjects. Using Pearson correlation tests, we found that the correlation between decoding accuracy and response time in EDS was -0.01 ($p = 0.92$) and the correlation between decoding accuracy and behavioral accuracy in EDS was 0.13 ($p = 0.32$). Given the lack of statistically significant findings, we have decided not to include these results into the main text.

2. In several figures (e.g. Fig 2, Fig 3c), the legend says that the image shows cluster corrected data at $p < 0.05$. However, the color bar indicates that the range is from $t > 2.0$, which is the t-value for $p < 0.05$, two-tailed for $N = 59$. This is an unusually low threshold for displaying fMRI results. It is more common to show data either at the

cluster-corrected threshold, or at a threshold of p < 0.01 or p < 0.001, uncorrected for "visualization purposes." In any case, the authors need to make it clearer which of these effects survive cluster correction, and also report the k-value used to threshold any surviving clusters. The manuscript is also missing tables summarizing statistical effects that survive cluster correction and their coordinates in MNI space, which would be very useful for other researchers wishing to follow up on these results, and for meta-analytic purposes.

Response: Thank you for highlighting this point. We would like to clarify that Figures 2, 3, 8, as well as S3, S5 and S7, all depict cluster-corrected results. Specifically, we applied a voxel-level threshold of $p < 0.05$ and excluded isolated voxels that did not survive a cluster-forming threshold of $p < 0.05$. In response to the reviewer's suggestion, we have now included the minimum cluster size (k) and neighborhood definition (NN=1, facewise neighbors) required to meet the corrected cluster-level threshold in the figure legends.

These details have been updated in "Materials and methods" section on Page 27, Line 12, "To correct for multiple comparisons, we used a whole-brain, family-wise error (FWE) cluster correction procedure (3dClustSim), where the smoothness was estimated using the updated spatial autocorrelation function option to reduce false positives (Cox et al., 2017). Clusters were formed by first applying a per-voxel p-value threshold of 0.05. This statistical significance of each cluster was determined using a cluster-level threshold of $p<0.05$, with a minimum cluster size (k) of 58 voxels to ensure that clusters reflect true positive signals while controlling for FWE across the whole brain."

In addition, we reported the center of mass (CM) coordinates for the activated clusters, and these results have been incorporated into the "Supporting information" S2.

3. In the decoding analysis (Figure 4), the authors test the performance of their decoder using a simple one-sample t-test against chance level performance. While this method was widely used in the early MVPA literature, it is considered overly liberal as the distribution of accuracy data from decoders do not meet the assumptions of the t-test against chance (see Stelzer et al., 2013, Neuroimage; Allefeld et al., 2016, Neuroimage). Please look to these papers and consider adopting a statistical approach that is more in line with known issues in analyzing these decoding accuracy data (e.g. non-parametric permutation tests).

Response: Thank you for your valuable suggestion. In response to this suggestion, we have implemented the non-parametric permutation test method as described by Stelzer et al. (2013). This approach provides more accurate statistical inferences by comparing our

empirical results to a null distribution generated through random permutations of the data, addressing concerns regarding the assumptions of t-tests in MVPA analyses. The null distribution also reflects any biases inherent in the data.

Following the examples outlined in Stelzer et al., we adopted a stringent voxel-level threshold of $p < 0.001$ and a cluster-level threshold of $p < 0.001$. The individual voxel p-values were determined using 10,000 bootstraps sampled from 500 random permutations for each subject, ensuring that each voxel has its own empirically derived threshold. Cluster sizes were determined based on the number of connected voxels (those exceeding the threshold), with connectivity defined as touching along an edge or face. These clusters were derived from the 10,000 bootstraps, yielding a null distribution of minimum cluster sizes. We are confident that this adjustment provides a more robust evaluation of our decoder's performance. Figure 4 has been updated to display the context decoding clusters that meet this stringent correction procedure.

And we have included a description of this method in the "Materials and methods" section on Page 28, Line 3, "To assess our findings at the group level, we compared the group average decoded results against an empirically determined null-distribution, using a cluster correction procedure to correct for multiple comparisons (Stelzer et al., 2013). The individual voxels were thresholded at $p < 0.001$, and only clusters that survived a cluster correction threshold of $p < 0.001$ were reported. The minimum cluster size was 343 voxels."

Stelzer, J., Chen, Y., & Turner, R. (2013). Statistical inference and multiple testing correction in classification-based multi-voxel pattern analysis (MVPA): random permutations and cluster size control. *Neuroimage*, *65*, 69-82.

4. **In the discussion, the authors claim that "Our results from MVPA analysis revealed that the thalamus preferentially encodes contextual information, but not other task-relevant information within the hierarchical task structure." I am afraid that this reads a bit like accepting the null finding that other task-relevant information is not encoded in the thalamus. This may not be the authors' intent, but I suggest they reword this to make it clear that they simply did not find evidence for such information being encoded in the thalamus, which they may have not had sufficient resolution or power to detect with fMRI.**

Response: Thank you for your feedback. We have revised the text on Page 21, Line 34, to clarify our findings not to affirm the null result: "Results from our MVPA analysis showed that the thalamus encodes contextual information. However, we did not find evidence that

other task-relevant information within the hierarchical task structure is encoded in the thalamus. This may be due to the limited resolution or statistical power of fMRI data to detect such signals.” Correspondingly, we have revised the conclusion of the decoding section in the “Abstract” on Page2, Line 9, “Decoding analysis revealed that thalamic activity encodes task contexts within the hierarchical task representations.”

5. It would be helpful if the authors provided more explanation of their null models for the activity flow analysis in the body of the results section.

Response: Thank you for your suggestion. We have expanded the explanation of our null models in the Results section as follows: Page 13, Line 7, “We then compared the performance of our model against two null models. The first null model assumed that no spatial pattern of thalamic evoked response carried task-related information. This was tested by randomly shuffling each subject’s thalamic evoked response vector with 5,000 random permutations. The second null model assumed that no thalamocortical functional connectivity pattern carried task-related information. This was tested by randomly shuffling elements within the FC matrix with 5,000 random permutations.”; and Page 15, Line 33, “We first compared our voxel-to-voxel thalamocortical interaction model to the two null models: the first assumed no spatial pattern of the thalamic evoked response carried task-related information, while the second assumed no thalamocortical functional connectivity pattern carried task-related information.”

6. In the methods section, the authors note that for their RSA analysis, "the neural dissimilarity was computed as one minus the spatial Mahalanobis distance." I think this must be a mistake, as this should only be done on correlation or cosine distances which are on a -1 to 1 scale, not on the Mahalanobis distance which is scaled only by the noise covariance.

Response: Thank you for pointing this out. We acknowledge that this was a misstatement in the “Materials and methods” section. In our analysis, we computed the neural dissimilarity directly using the Mahalanobis distance, without the “one minus” transformation, as the Mahalanobis distance is already scaled by the noise covariance. We have corrected the text in the methods section on Page 29, Line 41, “The neural dissimilarity was computed as the spatial Mahalanobis distance.”

7. The methods section mentions that no-smoothing was applied to the data. Is that true for all analyses? The univariate evoked responses (i.e. Figure 2) look as though some smoothing may have been applied. Please clarify where, if at all, smoothing was used - and the size of the smoothing kernel.

Response: Thank you for your question. We confirm that no spatial smoothing was applied to any of the neuroimaging data for all analyses. The appearance of smoothing in Figure 2 may be due to the visualization process rather than the data processing itself. We used the Nilearn package to project volumetric data onto the cortical surface, and this interpolation process can give the visual impression of smoothing. We incorporated this clarification into the “Materials and methods” section on Page 27, Line 18: “The visualization of cortical result was realized using Nilearn’s surface.vol_to_surf function, which projects the volumetric data onto the surface for display purpose.”

8. In Figure 7, it would be helpful to have a breakdown of the drop in prediction accuracy for removing voxels in different parts of the thalamus akin to panel e in Figure 5.

Response: Thank you for your suggestion. We have incorporated the reduction in prediction accuracy for cortical representations into Figure7.

9. The supplementary figures start at S4 rather than S1 and do not always correspond with the text. Please double-check and correct.

Response: Thank you for pointing it out. We apologize for the oversight. We have carefully reviewed and corrected the labeling of the supplementary materials.

10. Figure S7 (titled "Voxel-to-voxel BG-cortical model performance") was not clear to me. Are the top panels showing the results for predicting the data from ROIs identified by the RSA analyses for Context, Context x CO, etc.? I am not sure how these then relate to the task switching conditions (EDS, IDS, stay). Please provide more detail in the Figure legend explaining what the colors for each bar represent and what was being predicted.

Response: Thank you for pointing it out. We have revised the caption for Figure S9 (formerly S7), now titled “Voxel-to-voxel BG nuclei-cortical interaction model performances for predicting hierarchical task-switching conditions and cortical representations”.

Additionally, we have added the legends to the figure to clarify the different hierarchical-task switching conditions in S9a.

11. Please consider including individual data points in behavioral results in Figure 1c and d with lines connecting participants so that it is easier to visualize variability. Please also label error bars in the figure caption.

Response: Thank you for your suggestion. We have replotted the figure1c and 1d as requested, incorporating individual data points and lines connecting participants to visualize variability. And we have updated the figure1 captions to include a detailed explanation of the error bars.

12. Please consider making S5 (noise ceiling metrics) into a table. It is currently very hard to parse as a paragraph.

Response: Thank you for your suggestion. We have reformatted the noise ceiling results into tables, which can now be found in "Supporting information" S10.

Reviewer #3: This manuscript explores the role of the thalamus during a hierarchical decision making task involving frontal cognitive control. The authors collected fMRI data from ~60 healthy participants, and investigated how different hierarchical levels of task representation switch recruit the fronto-thalamic network. To do so, they used standard univariate analyses, decoding, as well as a more novel form of functional connectivity analysis, including simulated lesions in the thalamus. The results showed an increased activation in various thalamic nuclei especially when they had to make a rule based decision switch and that these activations were specific to updating at the highest hierarchical level of contextual representations. Finally, they found that these nuclei interact with other parts of the cortex involved in cognitive control, in particular parts of prefrontal cortex which replicate past findings.

This manuscript is relevant for the journal's scope and is original, it is overall well written and clear, and the results are original and relevant to the field. In general, the studies cited are appropriate, relevant and most of them are recent. The introduction contains all the relevant references and builds up the authors' hypotheses. The methods section provides essential information to understand the analysis performed. Showing cohen's d is good

practice to understand the impact of the results. The main results are well discussed. Overall, we found that this article is very solid and a useful addition to the literature on thalamic contributions to cognitive control. We note that we do not feel qualified to comment on the more intricate fMRI analyses.

Some points that could strength the manuscript

- Clarification on task structure. The methods don't clarify the randomization of the sequence of cues and stimuli. This is important as it controls the sequence of switches, which is at the core of the further analysis. Furthermore, there is no mention of how stay trials are balanced - in particular, there are two equivalent cues that allow for a stay trial to still be a "cue switch" (as in the figure example), but it is not clear whether all stay trials are "cue switch" trials or if some are also "cue stay" trials. Again, this could impact both the behavioral and imaging findings.

Response: Thank you for pointing this out. The sequence of cues and stimuli was fully randomized across trials. Cue repetition only occurred in the Stay condition, while no cue repetition was present in the EDS and IDS conditions. Cue repetition and cue switch trials were balanced. We have clarified this randomization has been clarified in the “Materials and methods” section on Page 25, Line 43, “The sequence of cues was fully randomized across trials, in which all 8 cues were randomly and equally distributed across trials.” Additionally, we have reported the number of trials for each condition in the “Materials and methods” section on Page 26, Line 7, “Each participant performed 204 Stay trials, 102 EDS trials, and 102 IDS trials.”

- Behavioral analysis. The analysis is quite minimal, looking only at EDS, IDS vs. stay. Cue stay vs. switch, and decision stay vs. switch are also known factors impacting RTs and accuracy, in ways that may interact with EDS and IDS. It seems important to analyze this in this context.

- These factors should also be considered within the fMRI analysis, in particular the initial univariate one and the decoding analyses. It is strange that the "decision" factor is considered in the later part of the paper but not the earlier part. It is clearly an important aspect of representations in this task.

Response: Thank you for this comment. We have incorporated the response repetition and cue repetition scenarios into both the behavioral results and neuroimaging results. These

results have been included in the “Results” section and “Supporting information” S1, S2, S3, S5 and S7.

Response repetition:

When analyzing trials where the response was either a repeat or not a repeat from the previous trial, our main findings remained unchanged: as the task-switching level increased from IDS to EDS trials, response time slowed, and accuracy decreased. This pattern was statistically significant and observed in both response repeat and response switch trials, indicating that our results are not driven by the response repeat factor. These results are described in detail on Page 5 Line 34:

“We next determined whether these effects were affected by repeating responses (choose “yes” or “no”) in the EDS and IDS conditions. Significant differences were found between EDS and IDS for both response switch (EDS vs. IDS: $t(58)=6.61$, $p<0.0001$, Cohen’s d =0.43; EDS vs. Stay: $t(58)=15.01$, $p<0.0001$, Cohen’s d =0.95; IDS vs. Stay: $t(58)=11.93$, $p<0.0001$, Cohen’s d =0.56) and response repeat (EDS vs. IDS: $t(58)=8.14$, $p<0.0001$, Cohen’s d=0.44; EDS vs. Stay: $t(58)=13.38$, $p<0.0001$, Cohen’s d =0.70; IDS vs. Stay: $t(58)=6.08$, $p<0.0001$, Cohen’s d =0.25; S1).”

A similar pattern was also observed in the univariate fMRI analysis, depicted in the “Supporting information” S3, S5 and S7.

Cue repetition:

There were no cue repeated scenarios in EDS and IDS conditions; cue repetition only occurred in the Stay condition. In the behavioral data, we did observe a significant difference between cue switch and cue repeat within Stay conditions, where cue switches (Stay_CS) were slower and less accurate than cue repeats (Stay_CR). We therefore tested whether differences between IDS and Stay trials is affected by cue repetition. Our results indicate that it is not, as described in detail in Page 5, Line 41. Specifically:

“We then examined whether task-switching effects were influenced by repeating cues in the Stay condition. Significant task-switching effects were observed when comparing both cue repeat (CR) and cue switch (CS) Stay trials for RT and accuracy. For RT, participants were significantly slower in the EDS condition compared to both Stay_CS ($t(58) = 12.99$, $p < 0.0001$, Cohen’s $d = 0.62$) and Stay_CR ($t(58) = 16.80$, $p < 0.0001$, Cohen’s $d = 0.97$; S1) trials. Similarly, participants were slower in the IDS condition compared to Stay_CS ($t(58) = 4.31$, $p = 0.001$, Cohen’s $d = 0.19$) and Stay_CR ($t(58) = 12.32$, $p < 0.0001$, Cohen’s $d = 0.55$). Additionally, participants responded more slowly in Stay_CS trials compared to Stay_CR

trials (*t*(58) = 7.42, $p < 0.0001$, Cohen's *d* = 0.36; S1). For accuracy, performance in the EDS condition was significantly lower than in Stay_CS (*t*(58) = -3.97, *p* = 0.002, Cohen's *d* = 0.30) and Stay_CR (*t*(58) = -8.01, p < 0.0001, Cohen's d = 0.70; S1) trials. In the IDS condition, accuracy was not significantly different from Stay_CS (*t*(58) = -0.125, *p* = 1.00, Cohen's *d* = 0.01) but was significantly lower than in Stay_CR (t(58) = -4.94, $p < 0.0001$, Cohen's *d* = 0.45). Furthermore, accuracy in Stay_CS trials was significantly lower than in Stay_CR trials (*t*(58) = -5.30, $p < 0.0001$, Cohen's *d* = 0.46; S1).

This suggests that the behavioral difference between IDS and Stay in our main findings is not driven by the cue repetition effect. Importantly, the neuroimaging results also remained consistent with our main findings when comparing IDS to either Stay_CS or Stay_CS trials, depicted in the "Supporting information" S3, S5 and S7.

Minor comments

P11 l1: FC matrix should be defined when first comes up, and it would be helpful to briefly say how it's derived to help understand the model within the main text.

Response: Thank you for pointing this out. We have spelled out FC at its first mention (Page 12, Line 27; formerly Page10, Line 25). Additionally, we have included an explanation of FC matrix in the main text (Page 12, Line 36; formerly Page11, Line 1): "Thalamocortical FC was calculated using principal component regression, which evaluates the functional connectivity between thalamus and cortices after regressing out stimuli-evoked signals, a measure also known as "background connectivity" (Cole et al., 2019)."

Fig. S1 is missing a legend for the line colors.

Response: Thank for pointing this out. We have included the missing legend in the updated Figure S4 (formerly S1) in "Supporting information" section.
